# Torque teno virus viremia and QuantiFERON^®^-CMV assay in prediction of cytomegalovirus reactivation in R+ kidney transplant recipients

**DOI:** 10.3389/fmed.2023.1180769

**Published:** 2023-06-22

**Authors:** Sarah Mafi, Marie Essig, Jean-Philippe Rerolle, Gisèle Lagathu, Romain Crochette, Véronique Brodard, Betoul Schvartz, Stephanie Gouarin, Nicolas Bouvier, Ilka Engelmann, Antoine Garstka, Céline Bressollette-Bodin, Diego Cantarovitch, Raphaële Germi, Benedicte Janbon, Christine Archimbaut, Anne-Elizabeth Heng, Françoise Garnier, Melissa Gomes-Mayeras, Anaïs Labrunie, Sébastien Hantz, Sophie Alain

**Affiliations:** ^1^French National Reference Center for Herpesviruses, Bacteriology, Virology, Hygiene Department, Centre Hospitalier Universitaire de Limoges, Limoges, France; ^2^Inserm, RESINFIT, U1092, Université de Limoges, Limoges, France; ^3^Nephrology and Transplantation Department, Centre Hospitalier Universitaire de Limoges, Limoges, France; ^4^Virology Department, Centre Hospitalier Universitaire de Rennes, Rennes, France; ^5^Nephrology and Transplantation Department, Centre Hospitalier Universitaire de Rennes, Rennes, France; ^6^Virology Department, Centre Hospitalier Universitaire de Reims, Reims, France; ^7^Nephrology and Transplantation Department, Centre Hospitalier Universitaire de Reims, Reims, France; ^8^Virology Department, Centre Hospitalier Universitaire de Caen, Caen, France; ^9^Nephrology and Transplantation Department, Centre Hospitalier Universitaire de Caen, Caen, France; ^10^Virology Department, Centre Hospitalier Universitaire de Lille, Lille, France; ^11^Nephrology and Transplantation Department, Centre Hospitalier Universitaire de Lille, Lille, France; ^12^Virology Department, Centre Hospitalier Universitaire de Nantes, Nantes, France; ^13^Nephrology and Transplantation Department, Centre Hospitalier Universitaire de Nantes, Nantes, France; ^14^Virology Department, Centre Hospitalier Universitaire de Grenoble, Grenoble, France; ^15^Nephrology and Transplantation Department, Centre Hospitalier Universitaire de Grenoble, Grenoble, France; ^16^Virology Department, Centre Hospitalier Universitaire de Clermont-Ferrand, Clermont-Ferrand, France; ^17^Nephrology and Transplantation Department, Centre Hospitalier Universitaire de Clermont-Ferrand, Clermont-Ferrand, France; ^18^Biostatistics Department, Centre Hospitalier Universitaire de Limoges, Limoges, France

**Keywords:** Torquetenovirus, cytomegalovirus, QuantiFERON^®^ CMV, kidney transplantation, CMV-seropositive recipients

## Abstract

**Introduction:**

Cytomegalovirus (CMV) is the most frequent infectious complication following solid organ transplantation. Torque teno viruses (TTV) viremia has been proposed as a biomarker of functional immunity in the management of kidney transplant recipients (KTR). The QuantiFERON^®^-CMV (QF-CMV) is a commercially available assay that allows the assessment of CD8^+^ T-cell responses in routine diagnostic laboratories.

**Methods:**

In a prospective national multicenter cohort of 64 CMV-seropositive (R+) KTR, we analyzed the value of TTV load and the two markers of the QF-CMV assay [QF-Ag (CMV-specific T-cell responses) and QF-Mg (overall T-cell responses)], alone and in combination, in prediction of CMV reactivation (≥3 log_10_ IU/ ml) in the first post-transplant year. We compared previously published cut-offs and specific cut-offs optimized from ROC curves for our population.

**Results:**

Using the conventional cut-off (3.45 log_10_ copies/ml), TTV load at D0 [inclusion visit on the day of transplantation before induction (D0)], or at M1 (1-month post-transplant visit) perform better in predicting CMV viremia control than CMV reactivation. Survival analyses suggest a better performance of our optimized TTV cut-offs (3.78 log_10_ copies/ml at D0 and 4.23 log_10_ copies/ml at M1) for risk stratification of CMV reactivation in our R+ KTR cohort. The QF-CMV (QF-Ag = 0.2 IU/ml, and QF-Mg = 0.5 IU/ml) also appears to better predict CMV viremia control than CMV reactivation. Moreover, survival analyses suggest that the QF-Mg would perform better than the QF-Ag in stratifying the risk of CMV reactivation. The use of our optimized QF-Mg cut-off (1.27 IU/ml) at M1 further improved risk stratification of CMV reactivation. Using conventional cut-offs, the combination of TTV load and QF-Ag or TTV load and QF-Mg did not improve prediction of CMV viremia control compared to separate analysis of each marker but resulted in an increase of positive predictive values. The use of our cut-offs slightly improved risk prediction of CMV reactivation.

**Conclusion:**

The combination of TTV load and QF-Ag or TTV load and QF-Mg could be useful in stratifying the risk of CMV reactivation in R+ KTR during the first post-transplant year and thereby have an impact on the duration of prophylaxis in these patients.

**Clinical trial registration:**

ClinicalTrials.gov registry, identifier NCT02064699.

## 1. Introduction

Cytomegalovirus (CMV) is the most common opportunistic viral infection and has a major impact on morbidity and mortality in solid organ transplant (SOT) recipients (1–3). Primary viral infection or reactivation of a latent CMV infection usually occurs in the first 6 months post-transplantation (1). CMV infection can result in both direct (CMV syndrome and tissue-invasive disease) and indirect effects (acute graft rejection, chronic graft failure, and opportunistic infections) (4). The incidence of CMV infection and CMV disease in SOT recipients depends on several factors, such as the CMV serological status of the donor (D) and the recipient (R), the type of organ transplant and the degree of immunosuppression (5). Current strategies for the prevention of CMV infection include prophylaxis (i.e., the use of antiviral drugs such as ganciclovir or valganciclovir during the 3–6 months post-transplantation) and pre-emptive therapy (i.e., the monitoring of CMV replication and administration of antiviral therapy solely in patients with CMV replication) (3). Preventive therapies have proven to be effective in reducing the risk of CMV infection and disease. Nevertheless, antiviral prophylaxis is associated with an increased risk of side effects compared with pre-emptive strategy especially neutropenia and nephrotoxicity. Drug toxicity can lead to premature discontinuation of treatment or reduced doses, resulting in non-response to treatment or emergence of resistance. Better targeting of prophylaxis duration based on reliable markers could thus be beneficial, especially for the lower-risk R+ recipients.

Specific cell-mediated immunity (CMI) response represents an essential host factor in the control of CMV infection (6). In recent years, several reports have investigated the potential clinical application of monitoring CMV-specific cellular immunity to better stratify the risk of CMV infection among recipients (7–12). Various CMV-specific immune-based assays have been developed to assess CMV specific T-cell responses. Intracellular cytokine staining (ICS) by flow cytometry can measure both virus-specific CD4^+^ and CD8^+^ T-cells but lacks technical standardization and is labor- and resource-intensive (3). The development of standardized CMV immune assays such as CMV enzyme-linked immunospot (ELISpot) assays and the QuantiFERON®-CMV (QF-CMV) assay has facilitated the assessment of CMV cell-mediated immunity in routine diagnostic laboratories (11). Although the ELISpot assay is highly sensitive, this assay cannot differentiate between CD4^+^ and CD8^+^ T-cell responses and requires PBMC isolation (3). The QF-CMV assay (Qiagen, Hilden, Germany) detects IFN-γ released in whole blood by CD8^+^ T-cells after *ex vivo* stimulation with a pool of 22 CMV short peptides presented by several human leukocyte antigen (HLA) class I haplotypes (13). This *in vitro* high-throughput diagnostic assay is simple to perform and has rapid turnaround times. Previous studies have demonstrated the potential utility of the QF-CMV in assessing the risk of CMV infection or disease in D+/R-SOT recipients or mixed populations (8, 14–19). In CMV-seropositive (R+) recipients, the QF-CMV assay appears to be a promising strategy to identify kidney transplant recipients (KTR) at highest or lowest risk of CMV infection or disease and, therefore, to tailor CMV prevention strategies to individual patients. Indeed, it would allow to avoid the severe infections inherent to the pre-emptive strategy and to decrease the neutropenic risk associated with the maintenance of a prophylactic treatment which is not essential if cell-mediated immunity is reconstituted.

More recently, Torque teno viruses (TTV) viremia has also been proposed as a biomarker of functional immunity in the management of transplant patients (20, 21). TTV, a member of the *Anelloviridae* family, is a small, single-stranded DNA virus and represents the most abundant virus of the human virome (22). Previous findings have shown that TTV viremia tends to be related to the level of immunosuppression (23–25) and its predictive value for viral infections is under evaluation (26–32). It has also been identified as a potential rejection predictor (30, 32–36) and a predictive marker of antibody response after COVID-19 vaccine in lung transplant recipients (37). Thus, monitoring TTV viremia could represent an additional diagnostic tool for prediction of CMV reactivation.

We conducted this study to evaluate the ability of TTV load and QF-CMV, alone and in combination, to predict CMV reactivation in R+ KTR during the first post-transplant year. We analyzed separately both markers of the QF-CMV assay to assess the impact of CMV-specific T-cell (QF-Ag) and overall T-cell (QF-Mg) responses. In addition to manufacturer’s recommendations, we refined TTV, QF-Ag, and QF-Mg cut-offs for risk stratification of CMV reactivation to guide optimal management of immunosuppressive drug doses in R+ KTR.

## 2. Materials and methods

### 2.1. Patient cohort and study design

The present multicenter, national, prospective cohort study, QuanticR+, was conducted from 2013 to 2017, with patients included from nine centers in France (Limoges, Clermont-Ferrand, Grenoble, Reims, Rennes, Lille, Caen, Nantes, and Besançon). A total of 73 CMV-seropositive patients (aged 18 years or older) who underwent kidney transplant were enrolled in this study. The protocol consisted of a pre-inclusion visit, an inclusion visit on the day of transplantation before induction (D0), and 9 other follow-up visits: monthly for the first 6 months (M1, M2, M3, M4, M5, and M6) and then every 2 months in the following 6 months (M8, M10, and M12) post-transplant (Supplementary Figure 1). Sixty-four patients (88%) completed a 1-year post-transplantation follow-up. Reasons for exclusion from the study included death (*n* = 1), graft rejection (*n* = 1), graft loss (*n* = 2), unwillingness of the patient to continue periodic monitoring visits (*n* = 2), and missing data (*n* = 3). At each visit, the QF-CMV assay was performed and CMV-DNA load in the blood was quantified by real-time PCR. Clinical data were collected by means of Electronic Case Report Forms (eCRFs). Peripheral blood samples were stored at −80°C in the biological collection of the Limoges University Hospital (CRBioLim, AC-2021-4790, certified NF S96-900) authorized by the French Ministry of Health. TTV-DNA load in peripheral blood was retrospectively quantified at the Herpesviruses National Reference Center. This study was approved by the local institutional review board (approval number: I10 002) and the Ethical Committee (16/04/2012, CPP SOOM IV), and registered at ClinicalTrials.gov registry (number: NCT02064699).

### 2.2. CMV serostatus

Pre-transplant CMV serostatus was determined by the specific anti-CMV IgG ELISA assay routinely used in each center’s laboratory.

### 2.3. CMV viral load quantification

The CMV-DNA real time PCR assays were routinely performed in centers according to the corresponding laboratory procedures. All centers participated annually to the international Quality Control for Molecular Diagnosis (QCMD). Assays results were reported in International Units per milliliter (IU/ml) as per the international World Health Organization (WHO) standard. For the centers who did not use the IU, positive samples were controlled with the National Reference Center assay (CMV R-GENE® kit, bioMérieux, France). CMV reactivation was defined as the presence of CMV-DNA level in blood ≥3 log_10_ IU/ml, a commonly used cut-off previously described in kidney recipients (38). For CMV syndrome and disease, definitions were developed specifically for transplant patients and were described in detail by Ljungman and colleagues (39). The CMV DNA cut-off chosen to start pre-emptive therapy was 3 log_10_ IU/ml.

### 2.4. TTV viral load quantification

Quantification of TTV-DNA viral load from whole blood was performed using the standardized TTV R-GENE® kit (bioMérieux, France), as specified by the manufacturer (40). Viral DNA was extracted from 200 μl EDTA whole blood using an easyMAG extractor (bioMérieux, France) with 140 μl of silica and a 50 μl elution volume. Ten μl of eluate was added to 15 μl of ready-to-use amplification premix (TTV R-GENE® assay, bioMérieux, France). In each run, an internal control and four quantification standards provided by the manufacturer were included. Amplification was performed according to manufacturer’s instructions on CFX96™ Real-Time PCR Detection System (Bio-rad Hercules, CA, USA). The limit of detection was 2.4 log_10_ copies/ml and the quantification range was between 2.4 to 9 log_10_ copies/ml.

### 2.5. QF-CMV assay

The QF-CMV assay (Qiagen, Hilden, Germany) was used in accordance with manufacturer’s instructions. Briefly, 1 ml of whole blood was collected into each of the three QF-CMV collection tubes: a CMV-antigen tube (QF-Ag) containing a mix of 22 CMV CD8^+^ T-cell synthetic epitopes, a mitogen tube (QF-Mg) containing phytohemagglutinin (positive control), and a nil tube containing only heparin (negative control). After an overnight incubation at 37°C, levels of IFN-γ (IU/ml) were measured by ELISA on plasma. As per manufacturer’s interpretive criteria, an IFN-γ value of ≥0.2 IU/ml in the QF-Ag tube (after subtraction of the IFN-γ level of the nil tube) was considered as a reactive result. The result was non-reactive if the QF-Ag minus the nil response was <0.2 IU/ml and the QF-Mg minus the nil response was >0.5 IU/ml. The test was reported as indeterminate when the QF-Ag minus the nil response was <0.2 IU/ml and the QF-Mg minus the nil response was <0.5 IU/ml.

### 2.6. Statistical analyses

Statistical analyses were conducted by the biostatistics unit (CDCR, University Hospital of Limoges) using SAS Enterprise Guide software version 7.1, Cary NC, USA. Significance level of *p*-value was 0.05. For descriptive analysis, continuous data were described as means ± standard deviation (SD) or median with data range (minimum to maximum, interquartile range), depending on the data distribution. Categorical variables were reported as number of patients and percentage. Differences between continuous variables were compared using the Mann–Whitney U test. The performance of TTV viremia and QF-Ag or QF-Mg for detection of CMV reactivation was assessed by calculating sensitivity, specificity, positive predictive value (PPV), and negative predictive value (NPV). Receiver operating characteristic (ROC) analyses were employed to determine optimal cut-off values of TTV viremia, QF-Ag, and QF-Mg for prediction of CMV reactivation. Survival curves modelling freedom from CMV reactivation ≥3 log_10_ IU/ml in the year post-transplant according to the result of TTV viremia, QF-Ag or QF-Mg were estimated using the Kaplan–Meier method. The hazard ratio with the associated 95% CI was calculated, and the log-rank test was used to compare Kaplan–Meier survival curves. The primary analysis was performed using a TTV cut-off of 3.45 log_10_ copies/ml that was previously identified by Maggi *et al*. at day 10 post-transplantation as a predictor of CMV viremia in the first 4 months post-transplantation (27), and manufacturer’s QF-CMV cut-offs (0.2 IU/ml and 0.5 IU/ml for the QF-Ag and the QF-Mg, respectively). The secondary analysis was conducted with optimal cut-offs determined by ROC curves. Since the QF-CMV assay cannot accurately measure IFN-γ values above 10 IU/ml, all values >10 IU/ml were treated as 10 IU/ml for the analyses.

## 3. Results

### 3.1. Demographic and clinical characteristics of patients

Patients median age was 54.4 ± 13.5 years with a slight majority of males (42/64; 65.6%) (Table 1). Most of the patients underwent a first transplantation (52/64; 81.3%). 28 out of 64 donors (43.8%) were CMV-seropositive and 45 patients (70.3%) received antiviral prophylaxis. The most commonly used antiviral prophylaxis was valganciclovir (41/45, 91.1%) at a dose of 900 mg (450 mg x 2) daily adjusted to renal function. The median prophylaxis time was 103.5 days, as recommended for R+ recipients. This duration was usually followed by clinicians from the study group but some variations were due to a shorter duration in case of graft loss (2 patients) or poor tolerance to the molecules, or a prolonged duration if the patient was considered at risk by the clinician.

**Table 1:** Characteristics of CMV-seropositive kidney transplant recipients included in the study.

Characteristics	All (*n* = 64)
Age (years; mean ± SD [min; max])	54.4 ± 13.5 [25; 78]
Gender, *n* (%)	
Female	22 (34.4%)
Male	42 (65.6%)
1^st^ transplantation, *n* (%)	52 (81.3%)
CMV serology status, *n* (%)	
D^+^/R^+^	28 (43.8%)
D^−^/R^+^	36 (56.3%)
Antiviral prophylaxis treatment, *n* (%)	45 (70.3%)
Valganciclovir/Ganciclovir	43 (95.6%)
Valaciclovir	2 (4.4%)
Duration of antiviral prophylaxis (days; median [IQR])	103.5 [86; 147.5]

SD, standard deviation; D, donor; R, recipient; and IQR, interquartile range.

Immunosuppressive regimen was quite homogeneous in all study centers. All patients except one received induction immunosuppression (basiliximab: 45/63 patients, thymoglobulin: 18/63 patients). 62/64 patients (data not available for 2/63 patients) received calcineurin inhibitors (CNI) as maintenance immunosuppression (tacrolimus 48/62 patients, ciclosporin: 14/62 patients) mainly combined with IMPDH inhibitors (53/64, missing data for 11 patients) and corticosteroids (62/64 patients). In patients without graft rejection, CNI exposition was progressively decreased during the first year (M0-M3: tacrolimus C0: 8–12 ng/ml, ciclosporin C2: 1000–1500 ng/ml; > M3: tacrolimus C0: 6–10 ng/ml, ciclosporin C2: 800–1200 ng/ml). Mycophenolate mofetil dosage was adjusted to reach a target AUC between 30–60 mg.h/L. Corticosteroids were tapered during the first weeks of the graft to a dose of 5–10 mg/d, and stopped at M3 in absence of rejection.

During the 1-year follow-up, 19 out of 64 patients (29.7%) [95% CI: 18.9; 42.4] developed CMV viremia ≥3 log_10_ IU/ml (Table 2). Of these, 3 patients developed CMV syndrome and a probable CMV colitis was reported in another patient. Six patients were treated with valganciclovir, 3 post-prophylaxis, and 3 as pre-emptive therapy. Median time to CMV reactivation in the cohort was 119 days [IQR: 63; 244] post-transplantation. Among the 45 patients who received prophylaxis, 13 developed CMV reactivation (28.9% [95% CI: 16.4; 44.3]), within a median of 143 days post-transplantation, consistent with discontinuation of antiviral prophylaxis. 6 of 19 patients without prophylaxis developed CMV reactivation (31.6% [95% CI: 11.6; 68.7]), within a median of 65 days post-transplantation.

**Table 2:** Distribution of the number of patients with CMV reactivation (≥ 3 log_10_ IU/ml) over the study periods.

Follow-up	CMV reactivation *n* (%) [95% CI]
D0–M6	14 (21.9%) [12.5; 34.0]
D0–M12	19 (29.7%) [18.9; 42.4]
M1–M4	11 (17.2%) [8.9; 28.7]
M1–M6	14 (21.9%) [12.5; 34.0]
M1–M12	19 (29.7%) [18.9; 42.4]

### 3.2. TTV viral load

#### 3.2.1. TTV viral load prevalence and dynamics

We investigated the kinetics of TTV viral load in our cohort to determine whether it could be a potential biomarker of interest for CMV reactivation.

**Figure 1:**
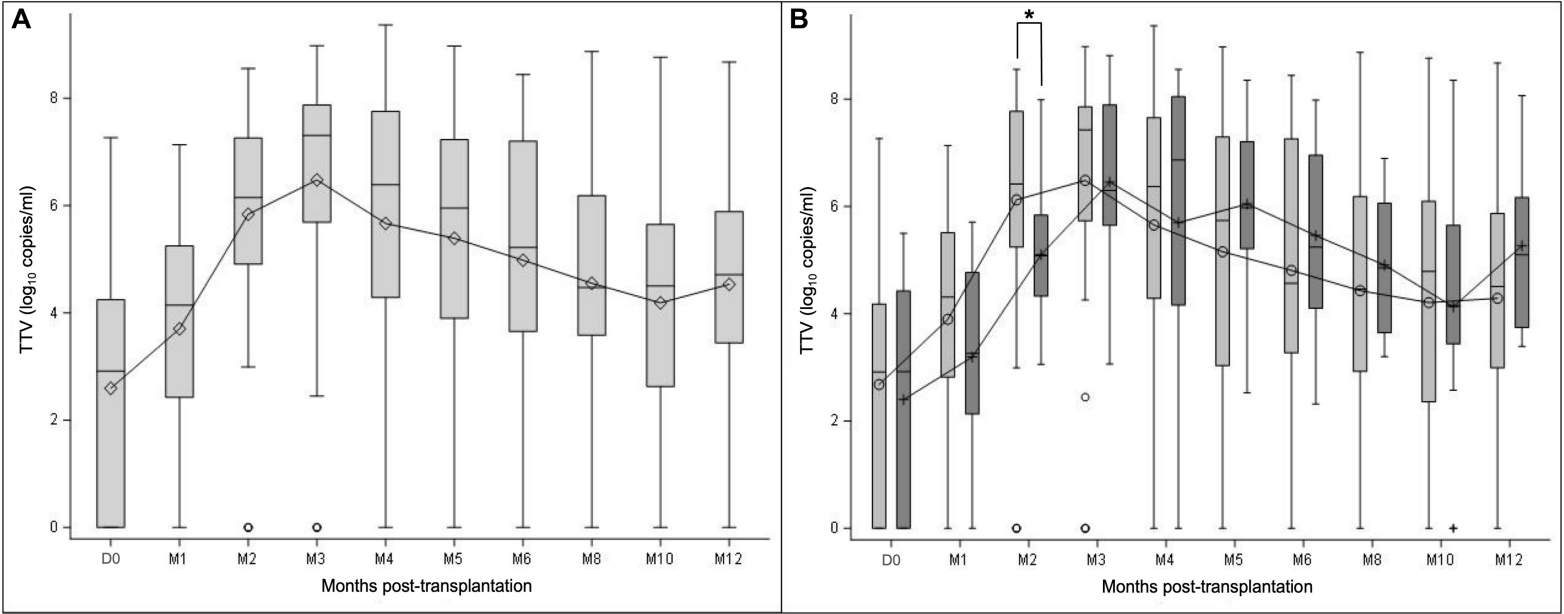
TTV load dynamics measured at transplantation (D0) and at month (M) 1, 2, 3, 4, 5, 6, 8, 10, and 12 after transplantation. The results are presented in the overall study population **(A)**, and in the subgroups of patients with (light grey) and without prophylaxis (dark grey) at D0 **(B)**. Mean values are displayed on the graph as points. Median values are represented by bars, and the interquartile range is represented by boxes. Significant differences are represented in the figure by an asterisk (*p* < 0.05).

TTV-DNA viremia ≥3.45 log_10_ copies/ml was detected in 44.1% of KTR at D0. TTV load kinetics showed an increasing phase from D0 (D0 mean TTV load: 2.6 log_10_ copies/ml) to M3 (M3 mean TTV load: 6.5 log_10_ copies/ml) (Figure 1A). After reaching a peak at M3, TTV load gradually decreased up to M12 (M12 mean TTV load: 4.5 log_10_ copies/ml).

TTV load kinetics were similar between subgroups of patients with and without prophylaxis (Figure 1B). No statistically significant difference in TTV load values at each time point was noted between these subgroups of patient (Mann–Whitney U test), except at M2 (*p* = 0.0070; Mann–Whitney U test).

#### 3.2.2. TTV viral load according to CMV reactivation

**Figure 2:**
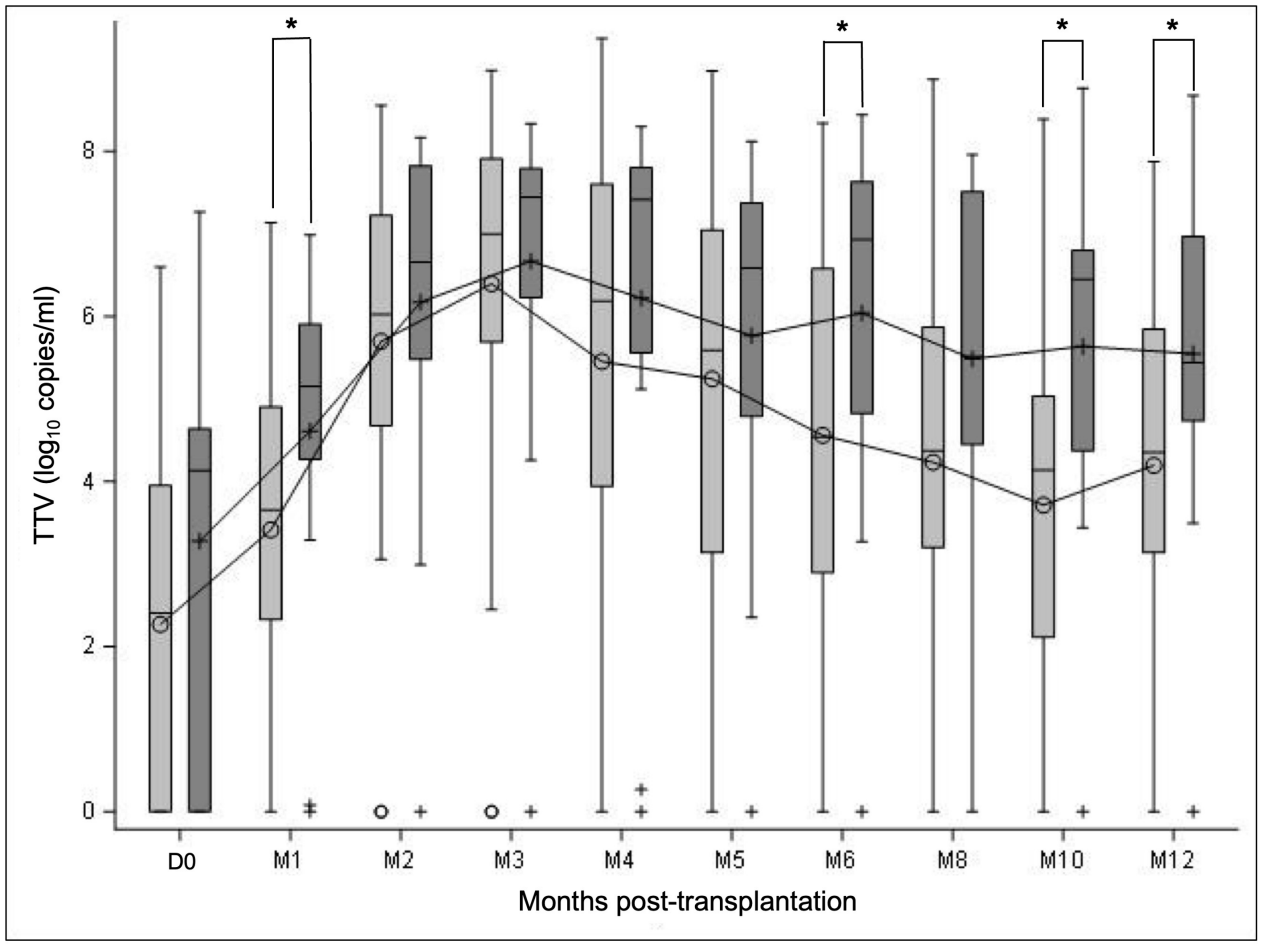
TTV load dynamics of all R+ KTR according to CMV reactivation. TTV load was measured at transplantation (D0) and at month (M) 1, 2, 3, 4, 5, 6, 8, 10, and 12 after transplantation. TTV kinetics are presented in the subgroups of patients with CMV reactivation ≥3 log_10_ IU/ml (dark grey) and without CMV reactivation (light gray) in the year post-transplant. Mean values are displayed on the graph as points. Median values are represented by bars, and the interquartile range is represented by boxes. Significant differences are represented in the figure by an asterisk (*p* < 0.05).

Dynamics of TTV DNAemia showed similar profiles in R+ KTR with and without CMV reactivation in the 1-year follow-up (Figure 2). Patients with CMV reactivation had higher TTV viral loads compared to patients without reactivation during the overall observation period. The observed difference between the subgroups of patients was statistically significant at M1 (*p* = 0.0297), M6 (*p* = 0.0424), M10 (*p* = 0.0207), and M12 (*p* = 0.0488; Mann–Whitney U test).

#### 3.2.3. Diagnostic accuracy of TTV load at D0 and M1 for prediction of CMV reactivation

We then evaluated the potential of peripheral TTV load at D0 and M1 for risk stratification of CMV reactivation using the cut-off proposed by Maggi et al. (3.45 log_10_ copies/ml) (Table 3).

**Table 3:** Diagnostic performance of TTV load using the previously determined cut-off (≥3.45 log_10_ copies/ml) for prediction of CMV reactivation in R+ KTR.

Cut-off (log_10_ copies/ml)	Time point	Follow-up	Sensitivity* %[95% CI]	Specificity* % [95% CI]	PPV* % [95% CI]	NPV* % [95% CI]
TTV ≥ 3.45	D0	D0–M6	57.1 [28.9; 82.3]	60.0 [44.3; 74.3]	30.8 [14.3; 51.8]	81.8 [64.5; 93.0]
	D0	D0–M12	63.2 [38.4; 83.7]	65.0 [48.3; 79.4]	46.2 [26.6; 66.6]	78.8 [61.1; 91.0]
	M1	M1–M4	75.0 [34.9; 96.8]	46.0 [31.8; 60.7]	18.2 [7.0; 35.5]	92.0 [74.0; 99.0]
	M1	M1–M6	72.7 [39.0; 94.0]	46.8 [32.1; 61.9]	24.2 [11.1; 42.3]	88.0 [68.8; 97.4]
	M1	M1–M12	78.6 [49.2; 95.3]	50.0 [34.6; 65.4]	33.3 [18.0; 51.8]	88.0 [68.8; 97.5]

PPV, positive predictive value; NPV, negative predictive value; and CI, confidence interval.

* Calculated in patients with data available over the study period.

A TTV load ≥3.45 log_10_ copies/ml at D0 yielded sensitivity of about 60%, specificity from 60 to 65%, PPV below 50%, and NPV of about 80% for CMV reactivation within the following 6 months (D0–M6) or 12 months (D0–M12). A TTV load ≥3.45 log_10_ copies/ml at M1 demonstrated sensitivity of at least 70%, specificity of about 50%, PPV below 40%, and NPV of at least 88% within the following 3 months (M1–M4), 5 months (M1–M6), or 11 months (M1–M12).

According to ROC curve analyses, the optimal TTV cut-offs at D0 were determined to be ≥3.97 log_10_ copies/ml (AUC = 0.56) and >3.78 log_10_ copies/ml (AUC = 0.65) for risk stratification of CMV reactivation within the following 6 and 12 months, respectively (Supplementary Figures 2A,B). These TTV cut-offs yielded sensitivity of about 60%, specificity of about 70%, PPV below 60%, and NPV of at least 80% within the following 6 or 12 months (Table 4).

**Table 4:** Diagnostic performance of TTV load using optimized cut-offs determined by ROC curves for prediction of CMV reactivation in R+ KTR.

Cut-off (log_10_ copies/ml)	AUC	Time point	Follow-up	Sensitivity* % [95% CI]	Specificity* % [95% CI]	PPV* % [95% CI]	NPV* % [95% CI]
TTV ≥ 3.97	0.56	D0	D0–M6	57.1 [28.9; 82.3]	71.1 [55.7; 83.6]	38.1 [18.1; 61.6]	84.2 [68.7; 94.0]
TTV > 3.78	0.65	D0	D0–M12	63.2 [38.4; 83.7]	72.5 [56.1; 85.4]	52.2 [30.6; 73.2]	80.6 [64.0; 91.8]
TTV ≥ 4.27	0.63	M1	M1–M4	75.0 [34.9; 96.8]	56.0 [41.2; 70.0]	21.4 [8.3; 40.9]	93.3 [77.9; 99.2]
TTV ≥ 4.27	0.63	M1	M1–M6	72.7 [39.0; 94.0]	57.4 [42.2; 71.7]	28.6 [13.2; 48.7]	90.0 [73.5; 97.9]
TTV > 4.23	0.70	M1	M1–M12	78.6 [49.2; 95.3]	61.4 [45.5; 75.6]	39.2 [21.5; 59.4]	90.0 [73.5; 97.9]
TTV > 0.75	0.60	D0 to M1	M1–M12	78.6 [49.2; 95.3]	48.7 [32.4; 65.2]	35.5 [35.5; 19.2]	86.4 [65.1; 97.1]

AUC, Area under the ROC Curve; PPV, positive predictive value; NPV, negative predictive value; and CI, confidence interval.

* Calculated in patients with data available over the study period.

The optimal TTV cut-offs at M1 were found to be ≥4.27 log_10_ copies/ml (AUC = 0.63) for risk stratification of CMV reactivation within the following 3 or 5 months, and >4.23 log_10_ copies/ml (AUC = 0.70) for risk stratification of CMV reactivation within the following 11 months (Supplementary Figures 2D–F). These TTV cut-offs yielded sensitivity between 70 and 80%, specificity of about 60%, PPV below 40%, and NPV of at least 90% within the following 3, 5, or 11 months (Table 4).

Dynamic changes in TTV levels between D0 and M1 were also assessed as a predictor of CMV reactivation within the following 11 months (M1-M12) (Supplementary Figure 2C). An increase of at least 0.75 log_10_ copies/ml from D0 to M1 (AUC = 0.60) demonstrated a sensitivity of 79%, a specificity of 49%, a PPV of 36%, and a NPV of 86% (Table 4).

#### 3.2.4. Kaplan–Meier survival analyses

**Figure 3:**
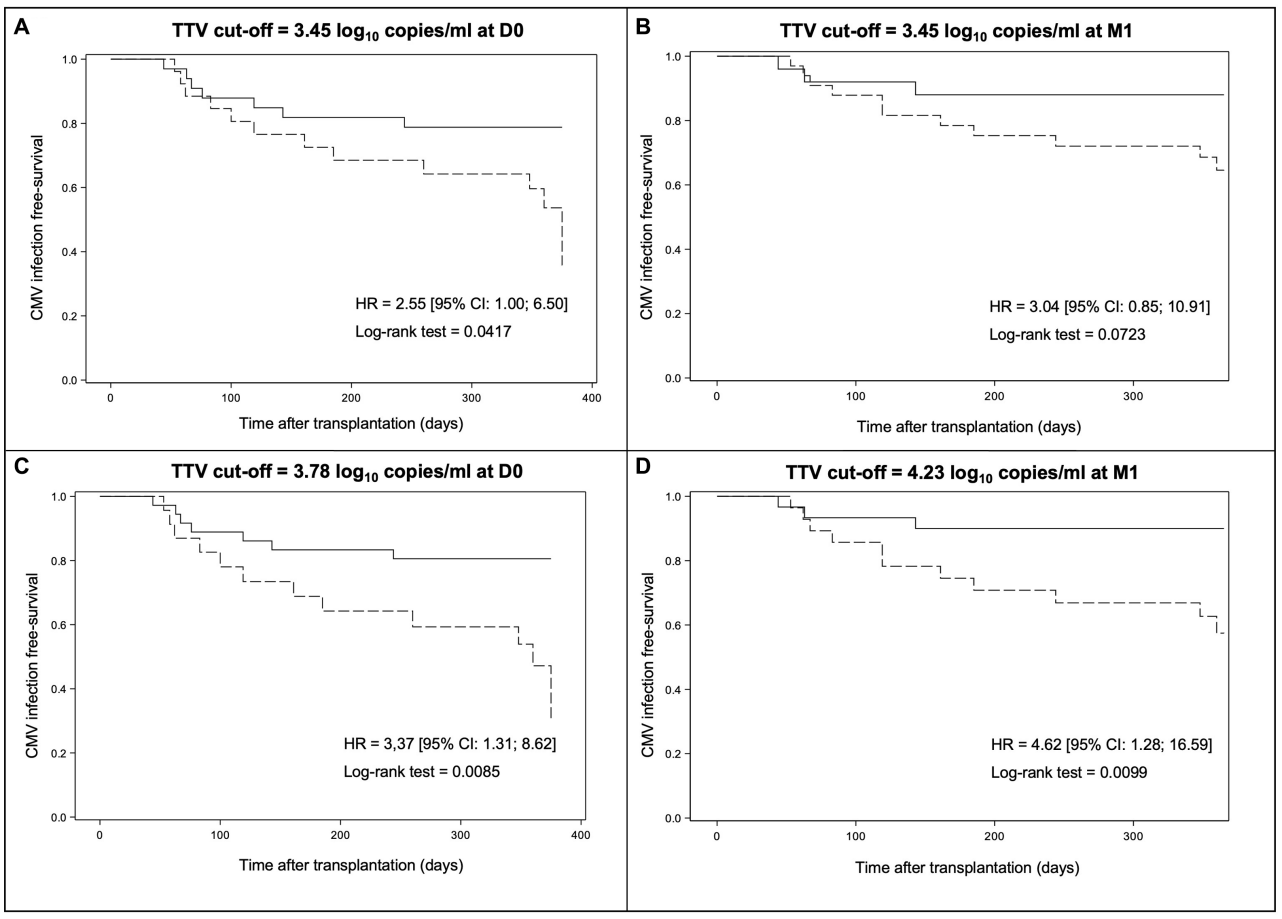
Kaplan–Meier survival curves modelling freedom from CMV reactivation through day + 360 post-transplant according to the result of TTV load at D0 or M1. The results are presented according to the TTV cut-off of 3.45 log_10_ copies/ml at D0 **(A)**, the TTV cut-off of 3.45 log_10_ copies/ml at M1 **(B)**, the optimal TTV cut-off at D0 determined by ROC curves (3.78 log_10_ copies/ml) **(C)**, and the optimal TTV cut-off at M1 as defined by ROC curves (4.23 log_10_ copies/ml) **(D)**. Dotted lines indicate TTV load above the cut-off while solid lines indicate TTV load below the cut-off. HR, hazard ratio and CI, confidence interval.

**Figure 4:**
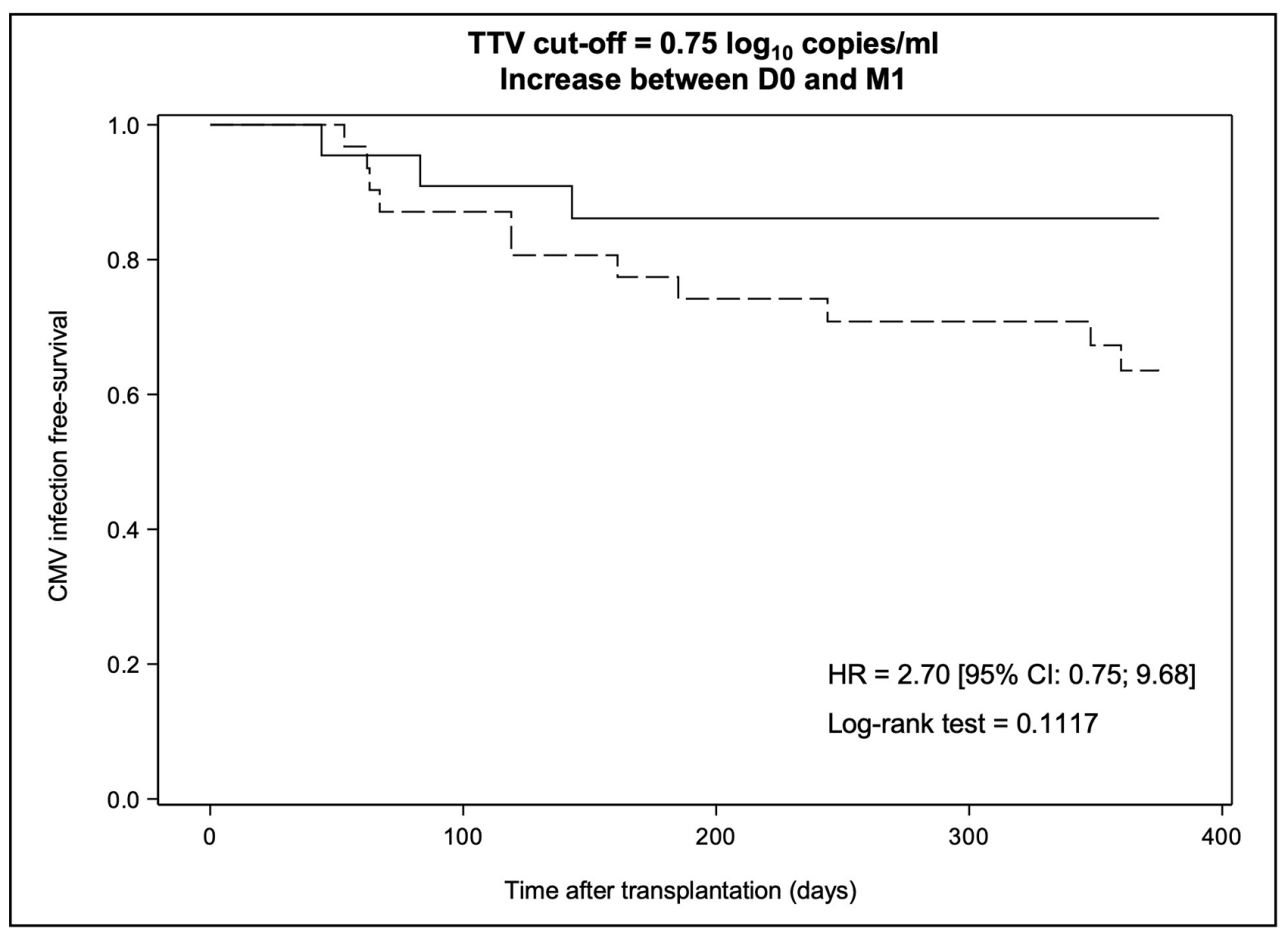
Kaplan–Meier survival curves modelling freedom from CMV reactivation through day + 360 post-transplant according to the dynamic change in TTV levels between D0 and M1. The dotted line indicates an increase in TTV levels >0.75 log_10_ copies/ml while the solid line indicates a TTV dynamic change below this value. HR, hazard ratio and CI, confidence interval.

The cumulative incidence of CMV reactivation in the year post-transplant according to the result of TTV load is shown in Figures 3 and 4. The analysis was performed using the TTV cut-off proposed by Maggi et al. (3.45 log_10_ copies/ml) at D0 and M1, the optimal TTV cut-offs determined by ROC curves (3.78 log_10_ copies/ml at D0 and 4.23 log_10_ copies/ml at M1), and the optimal increase in TTV levels between D0 and M1 (0.75 log_10_ copies/ml).

By using the TTV cut-off of 3.45 log_10_ copies/ml, a significant difference in CMV infection-free survival at M12 was observed between patients with a TTV load above or below the cut-off at D0 (35.8% [95% CI: 8,7; 64,9] versus 78.8% [95% CI: 60,6; 89,3], respectively, *p_log-rank_* = 0.0417) but not at M1 (64.6% [95% CI: 44.9; 78.7] versus 88.0% [95% CI: 67.3; 96.0], respectively, *p_log-rank_* = 0.0723) (Figures 3A,B). Thus, a TTV load ≥3.45 log_10_ copies/ml at D0 predicts a significantly higher risk of CMV reactivation than a TTV load <3.45 log_10_ copies/ml in our R+ KTR cohort. However, this cut-off at M1 does not predict a significant higher risk of CMV reactivation in our R+ KTR cohort.

By using the TTV cut-off of 3.78 log_10_ copies/ml, Kaplan–Meier analysis revealed a significant difference in CMV infection-free survival at M12 between patients with a TTV load above or below the cut-off at D0 (23.6% [95% CI: 1.9; 59.2] versus 80.6% [95% CI: 63.5; 90.2], respectively, *p_log-rank_* = 0.0085) (Figure 3C). By using the TTV cut-off of 4.23 log_10_ copies/ml, a significant difference in CMV infection-free survival at M12 between patients with a TTV load above or below the cut-off at M1 was also obtained (57.5% [95% CI: 35.9; 74.1] versus 90.0% [95% CI: 72.1; 96.7], respectively, *p_log-rank_* = 0.0099) (Figure 3D). In our R+ KTR cohort, a TTV viremia >3.78 log_10_ copies/ml at D0 or >4.23 log_10_ copies/ml at M1 therefore predicts a significantly higher risk of CMV reactivation.

No significant difference in CMV infection-free survival at M12 was observed between patients with an increase in TTV levels of at least 0.75 log_10_ copies/ml from D0 to M1 and the remaining group (63.5% [95% CI: 43.8; 78.0] versus 86.1% [95% CI: 62.9; 95.3], respectively, *p_log rank_* = 0.1117) (Figure 4). Thus, an increase in TTV levels of at least 0.75 log_10_ copies/ml from D0 to M1 does not predict a significantly higher risk of CMV reactivation.

### 3.3. QF-CMV assay

#### 3.3.1. Kinetics of CD8^+^ T-cell responses

**Figure 5:**
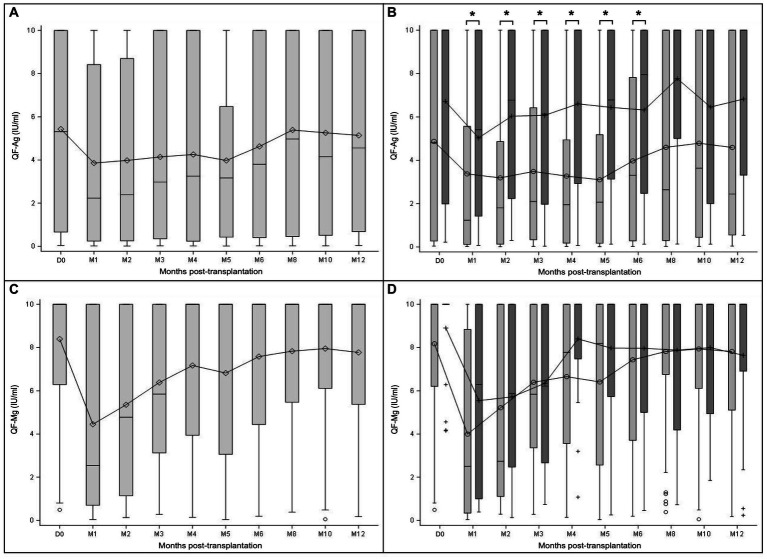
QF-CMV assay dynamics measured at transplantation (D0) and at month (M) 1, 2, 3, 4, 5, 6, 8, 10, and 12 after transplantation. The results are presented separately for the QF-Ag and the QF-Mg in the overall study population **(A,C)**, and in the subgroups of patients with (light grey) and without prophylaxis (dark grey) at D0 **(B,D)**. Mean values are displayed on the graph as points. Median values are represented by bars, and the interquartile range is represented by boxes. Significant differences are represented in the figure by an asterisk (*p* < 0.05).

Specific CD8^+^ T-cell responses are crucial to control CMV reactivation. We followed their kinetics using the QF-Ag. At engraftment (J0), 85.7% of patients (54/63 patients) had a reactive QF-Ag indicating the presence of CMV-specific CD8^+^ T cells (Figure 5). A strong decrease was observed in QF-Ag responses between D0 and M1 (D0: 5.4 IU/ml, M1: 3.9 IU/ml), followed by a plateau phase between M1 and M5, with mean values ranging from 3.9 to 4.3 IU/ml and an increase after M5 (Figure 5A).

Regarding global CD8^+^ T-cell responses according to the QF-Mg, 98.4% of patients (62/63) responded to mitogen at D0. After a substantial initial decline from baseline to M1 (D0: 8.4 IU/ml, M1: 4.4 IU/ml), the mean values increased until reaching 7.2 IU/ml at M4 and 6.8 IU/ml at M5 (Figure 5C). Then, QF-Mg values remained relatively stable between M6 and M12 (M6: 7.6 IU/ml, M8: 7.8 IU/ml, M10: 8.0 IU/ml, M12: 7.8 IU/ml).

Interestingly, in patients receiving prophylaxis, CMV-specific CD8^+^ T-cell responses (mean QF-Ag values) were significantly lower from M1 to M6 compared to patients without prophylaxis (M1 *p* = 0.0112, M2 *p* = 0.0459, M3 *p* = 0.0057, M4 *p* = 0.0053, M5 *p* = 0.0377, M6 *p* = 0.0404; Mann–Whitney U test) (Figure 5B). In addition, CMV-specific CD8^+^ T-cell dynamics results showed a first increase between M1 and M2 in patients without prophylaxis, while a first increase occurred only from M5 in the subgroup of patients with prophylaxis. Dynamic changes in global CD8^+^ T-cell responses (QF-Mg values) were broadly similar between subgroups of patients with and without prophylaxis (Figure 5D). There was no statistically significant difference in QF-Mg values between patients with and without prophylaxis during the follow-up year (Mann–Whitney U test). These results suggest that in the absence of prophylaxis, patients have a higher number of CMV-specific CD8^+^ T cells.

#### 3.3.2. CD8^+^ T-cell responses according to CMV reactivation

**Figure 6:**
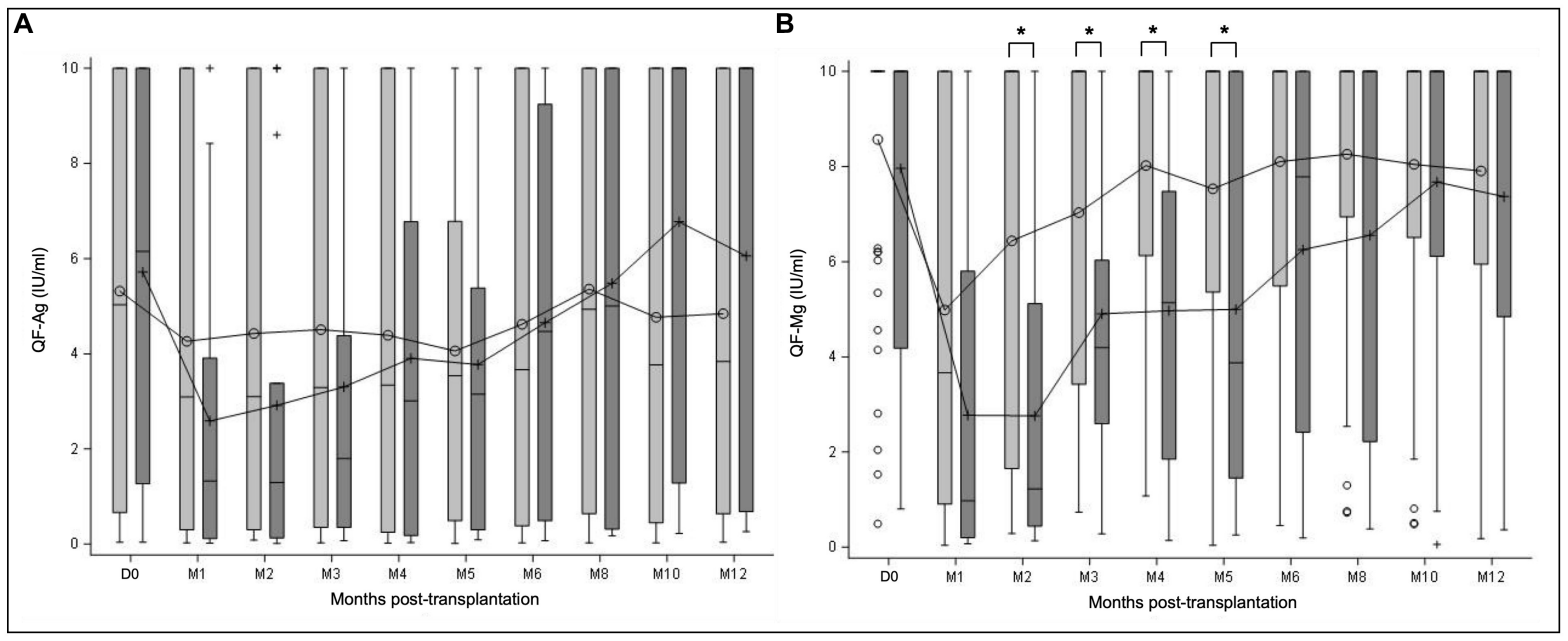
QF-CMV assay dynamics of all R+ KTR according to CMV reactivation. QF-assay was measured at transplantation (D0) and at month (M) 1, 2, 3, 4, 5, 6, 8, 10, and 12 after transplantation. QF-Ag **(A)** and QF-Mg **(B)** kinetics are presented in the subgroups of patients with CMV reactivation ≥3 log_10_ IU/ml (dark grey) and without CMV reactivation (light grey), as measured in the year post-transplant. Mean values are displayed on the graph as points. Median values are represented by bars, and the interquartile range is represented by boxes. Significant differences are represented in the figure by an asterisk (*p* < 0.05).

Then, we aimed to determine if CMV viremia was associated with CMV-specific or global CD8^+^ T-cell kinetics. To do so, we analyzed QF-Ag and QF-Mg kinetics in patients who experienced CMV reactivation during the 1-year follow-up compared to those who had CMV viremia <3 log_10_ IU/ml (Figure 6). Except at D0, mean QF-Ag values were lower in patients with CMV reactivation during the first 5 months after transplantation (Figure 6A), although it failed to reach statistical significance (Mann–Whitney U test). In patients with CMV reactivation, mean QF-Ag values gradually increased until reaching mean QF-Ag values of patients without reactivation at M6. From M8 to M12, mean QF-Ag values tended to be higher in the subgroup of patients with CMV reactivation.

In the same way as for the QF-Ag, mean QF-Mg values at D0 were similar between patients with and without CMV reactivation. From M1 to M12, QF-Mg mean values were lower in patients with CMV reactivation, compared to patients without CMV reactivation (Figure 6B). Of note, this trend approached the level of significance at M1, and achieved statistical significance between M2 and M5 (M1 *p* = 0.0501, M2 *p* = 0.0009, M3 *p* = 0.0496, M4 *p* = 0.0019, M5 *p* = 0.0312; Mann–Whitney U test). In KTR without CMV reactivation, the QF-Mg kinetics revealed an increasing phase between M1 and M4, followed by a plateau phase until M12, while QF-Mg values increased progressively from M1 to M12 in patients with CMV reactivation.

These results demonstrate that CMV-specific CD8^+^ T-cell responses are not statistically different in patients with CMV reactivation compared to patients without CMV reactivation. In contrast, patients with CMV reactivation have significantly lower overall T-cell responses between M2 and M5.

#### 3.3.3. Diagnostic accuracy of the QF-Ag or the QF-Mg at M1 for prediction of CMV reactivation

To determine whether the QF-Ag or the QF-Mg at M1 could be potential biomarkers for CMV reactivation within the following 3 months (M1-M4), 5 months (M1-M6), and 11 months (M1-M12), sensitivity, specificity, PPV and NPV were calculated.

The primary analysis was performed using manufacturer’s cut-offs (0.2 and 0.5 IU/ml for the QF-Ag and the QF-Mg, respectively) (Table 5). The sensitivity was no more than 30% for the QF-Ag and less than 50% for the QF-Mg, whatever the follow-up period studied. In contrast, both markers had better specificity with values of at least 78% for the QF-Ag and 80% for the QF-Mg. The QF-Ag allowed the identification of at least 77% of patients without CMV reactivation. Likewise, the QF-Mg displayed excellent NPV with values of at least 82%. However, PPV were low for the QF-Ag (< 40%) and the QF-Mg (< 50%).

**Table 5:** Diagnostic performance of the QF-Ag or the QF-Mg at M1 using manufacturer’s cut-offs for prediction of CMV reactivation in the R+ KTR cohort.

Cut-off (IU/ml)	Time point	Follow-up	Sensitivity* % [95% CI]	Specificity* % [95% CI]	PPV* % [95% CI]	NPV* % [95% CI]
QF-Ag < 0.2	M1	M1–M4	25.0 [3.2; 65.1]	78.0 [64.0; 88.5]	15.4 [1.9; 45.4]	86.7 [73.2; 95.0]
	M1	M1–M6	27.3 [6.0; 61]	78.7 [64.3; 89.3]	23.1 [5.0; 53.8]	82.2 [68.0; 92.0]
	M1	M1–M12	28.6 [8.4; 58.1]	79.5 [64.7; 90.2]	30.8 [9.1; 61.4]	77.8 [62.9; 88.8]
QF-Mg < 0.5	M1	M1–M4	37.5 [8.5; 75.5]	80.0 [66.3; 90.0]	23.1 [5.0; 53.8]	88.9 [76.0; 96.3]
	M1	M1–M6	45.5 [16.8; 76.6]	83.0 [69.2; 92.4]	38.5 [13.9; 68.4]	86.7 [73.2; 95.0]
	M1	M1–M12	42.9 [17.7; 71.1]	84.1 [69.9; 93.4]	46.1 [19.2; 74.9]	82.2 [67.9; 92.0]

PPV, positive predictive value; NPV, negative predictive value; and CI, confidence interval.

* Calculated in patients with data available over the study period.

Receiving operator curve (ROC) analyses were used to define QF-Ag and QF-Mg cut-offs at M1 that would best discriminate patients with and without subsequent CMV reactivation. For the QF-Ag at M1, the optimal cut-offs were determined to be <2.23 IU/ml (AUC = 0.53), < 9.12 IU/ml (AUC = 0.56) and ≤ 2.48 IU/ml (AUC = 0.61) to predict CMV reactivation within the following 3, 5, and 11 months, respectively (Supplementary Figures 3A–C). The optimal QF-Mg cut-offs at M1 were found to be <1.29 IU/ml (AUC = 0.61), < 1.29 IU/ml (AUC = 0.69), and ≤ 1.27 IU/ml (AUC = 0.68) to predict CMV reactivation within the following 3, 5, and 11 months, respectively (Supplementary Figures 3D–F).

By using these optimized cut-offs, higher sensitivity was obtained with values ranging from 63 to 91% for the QF-Ag, and ranging from 63 to 73% for the QF-Mg (Table 6). Specificity was between 28 and 55% for the QF-Ag, and about 70% for the QF-Mg. A slighter increase of NPV was obtained for the QF-Ag and the QF-Mg with values of at least 86%, whereas PPV were low (<50%).

**Table 6:** Diagnostic performance of the QF-Ag or the QF-Mg at M1 using optimized cut-offs determined by ROC curves for prediction of CMV reactivation in the R+ KTR cohort.

Cut-off (IU/ml)	AUC	Time point	Follow-up	Sensitivity* % [95% CI]	Specificity* % [95% CI]	PPV* % [95% CI]	NPV* % [95% CI]
QF-Ag < 2.23	0.53	M1	M1–M4	62.5 [24.5; 91.5]	52.0 [37.4; 66.3]	17.2 [5.8; 35.8]	89.7 [72.7; 97.8]
QF-Ag < 9.12	0.56	M1	M1–M6	90.9 [58.7; 99.8]	27.7 [15.6; 42.6]	22.7 [11.5; 37.8]	92.9 [66.1; 99.8]
QF-Ag ≤ 2.48	0.61	M1	M1–M12	71.4 [41.9; 91.6]	54.5 [38.8; 69.6]	33.3 [17.3; 52.8]	85.7 [67.3; 95.6]
QF-Mg < 1.29	0.61	M1	M1–M4	62.5 [24.5; 91.5]	68.0 [53.3; 80.5]	23.8 [8.2; 47.2]	91.9 [78.1; 98.3]
QF-Mg < 1.29	0.69	M1	M1–M6	72.7 [39.0; 94.0]	72.3 [57.4; 84.4]	38.1 [18.1; 61.6]	91.9 [78.1; 98.3]
QF-Mg ≤ 1.27	0.68	M1	M1–M12	64.3 [35.1; 87.2]	72.7 [57.2; 85.0]	42.9 [21.8; 66.0]	86.5 [71.2; 95.5]

AUC, Area under the ROC Curve; PPV, positive predictive value; NPV, negative predictive value; and CI, confidence interval.

* Calculated in patients with data available over the study period.

#### 3.3.4. Kaplan–Meier survival analyses

Subsequently, we investigated the cumulative incidence of CMV reactivation in the year post-transplant according to the result of the QF-CMV. The analysis was performed separately for the QF-Ag and the QF-Mg using manufacturer’s cut-offs (0.2 and 0.5 IU/ml, respectively) and optimal cut-offs determined by ROC curves (2.48 and 1.27 IU/ml, respectively).

**Figure 7:**
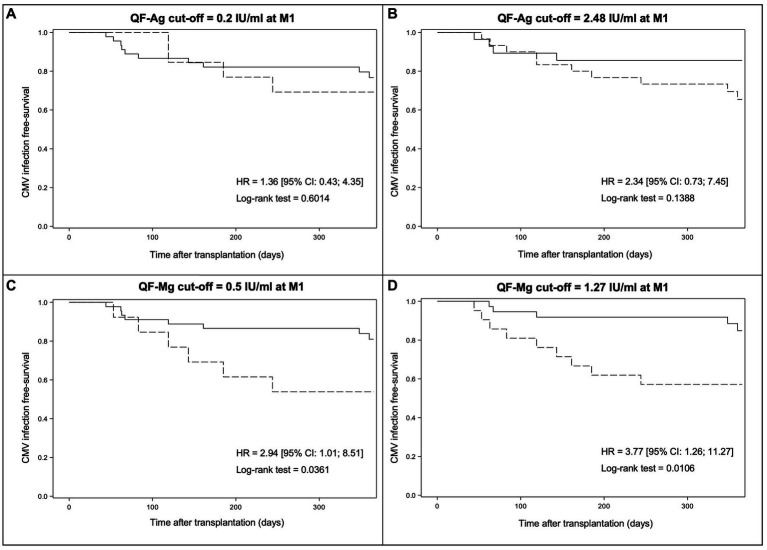
Kaplan–Meier survival curves modelling freedom from CMV reactivation from M1 to M12 according to the result of the QF-CMV at M1. The results are presented for the manufacturer’s QF-Ag cut-off **(A)**, the optimal QF-Ag cut-off determined by ROC curves **(B)**, the manufacturer’s QF-Mg cut-off **(C)**, and the optimal QF-Mg cut-off determined by ROC curves **(D)**. Solid lines indicate QF-CMV results above the cut-off while dotted lines indicate QF-CMV results below the cut-off. HR, hazard ratio and CI, confidence interval.

Using the 0.2 IU/ml cut-off, no significant difference in CMV infection-free survival at M12 was observed between patients with a reactive and a non-reactive QF-Ag at M1 (76.7% [95% CI: 60.8; 86.8] versus 69.2% [95% CI: 37.3; 87.1], respectively, *p_log-rank_* = 0.6014) (Figure 7A). Likewise, we did not observe a significant difference in CMV infection-free survival at M12 using the 2.48 IU/ml cut-off (85.6% [95% CI: 66.0; 94.3] versus 65.4% [95% CI: 45.0; 79.8], respectively, *p_log-rank_* = 0.1388) (Figure 7B).

Conversely, Kaplan–Meier analysis revealed a significant difference in CMV infection-free survival at M12 between patients with a reactive and a non-reactive QF-Mg at M1 (80.9% [95% CI: 65.2; 90.0] versus 53.8% [95% CI: 24.8; 76.0], respectively, *p_log-rank_* = 0.0361) using the 0.5 IU/ml cut-off (Figure 7C). A stronger difference was observed using the 1.27 IU/ml cut-off (84.8% [95% CI: 67.0; 93.5] versus 57.1% [95% CI: 33.8; 74.9], respectively, *p_log-rank_* = 0.0106)(Figure 7D). Altogether, these results indicated that a cut-off of 1.27 IU/ml allows a better stratification of CMV reactivation risk.

### 3.4. TTV viral load in combination with the QF-Ag or the QF-Mg

The combination of TTV load ≥3.45 log_10_ copies/ml and the QF-Ag < 0.2 IU/ml at M1 was assessed for prediction of CMV reactivation within the following 3 months (M1-M4), 5 months (M1-M6), and 11 months (M1-M12) in R+ KTR (Table 7). Sensitivity did not exceed 30% whatever the follow-up period studied, while specificity was of at least 85%. High NPV were obtained (> 79%), whereas PPV were below 50%.

**Table 7:** Diagnostic performance of the QF-Ag or QF-Mg and TTV load analyzed in combination at M1 for prediction of CMV reactivation in R+ KTR.

Cut-off (QF-CMV: IU/ml, TTV: log_10_ copies/ml)	Time point	Follow-up	Sensitivity* % [95% CI]	Specificity* % [95% CI]	PPV* % [95% CI]	NPV* % [95% CI]
QF-Ag < 0.2 and TTV ≥ 3.45	M1	M1–M4	25.0 [3.2; 65.0]	85.7 [72.8; 94.1]	22.2 [2.8; 60.0]	87.5 [74.7; 95.3]
	M1	M1–M6	27.3 [6.0; 61.0]	87.0 [73.7; 95.1]	33.3 [7.5; 70.1]	83.3 [70.0; 92.5]
	M1	M1–M12	28.6 [8.4; 58.1]	88.4 [74.9; 96.1]	44.4 [13.7; 78.8]	79.2 [65.0; 89.5]
QF-Mg < 0.5 and TTV ≥ 3.45	M1	M1–M4	37.5 [8.5; 75.5]	89.8 [77.8; 96.6]	37.5 [8.5; 75.5]	89.8 [77.8; 96.6]
	M1	M1–M6	36.4 [10.9; 69.2]	91.3 [79.2; 97.6]	50.0 [15.7; 84.3]	91.3 [72.8; 94.1]
	M1	M1–M12	35.7 [12.8; 64.9]	93.0 [80.9; 98.5]	62.5 [24.5; 91.5]	81.6 [68.0; 91.2]

The diagnostic accuracy of the combination of markers was assessed using QF-CMV manufacturer’s cut-offs and the previously determined TTV cut-offs. PPV, positive predictive value; NPV, negative predictive value; and CI, confidence interval.

* Calculated in patients with data available over the study period.

The combination of TTV load ≥3.45 log_10_ copies/ml and the QF-Mg < 0.5 IU/ml at M1 for detection of CMV reactivation was also analyzed (Table 7). Sensitivity values were about 35%, while specificity reached values of about 90%. This combination exhibited high NPV (> 80%), whereas PPV ranged from 38 to 63%.

Using optimized cut-offs, TTV load in combination with the QF-Ag demonstrated sensitivity ranging from 50 to 64%, and specificity from 70 to 86% (Table 8). High NPV were obtained (> 88%), whereas PPV ranged from 29 to 60%.

**Table 8:** Diagnostic performance of the QF-Ag or QF-Mg and TTV load analyzed in combination at M1 for prediction of CMV reactivation in R+ KTR.

Cut-off (QF-CMV: IU/ml,TTV: log_10_ copies/ml)	Time point	Follow-up	Sensitivity* % [95% CI]	Specificity* % [95% CI]	PPV* % [95% CI]	NPV* % [95% CI]
QF-Ag < 2.23 and TTV ≥ 4.27	M1	M1–M4	50.0 [15.7; 84.3]	79.6 [65.7; 89.8]	28.6 [8.4; 58.1]	90.7 [77.9; 97.4]
QF-Ag < 9.12 and TTV ≥ 4.27	M1	M1–M6	63.6 [30.8; 89.1]	69.6 [54.2; 82.3]	33.3 [14.6; 57.0]	88.9 [73.9; 97.0]
QF-Ag ≤ 2.48 and TTV > 4.23	M1	M1–M12	64.3 [35.1; 87.2]	86.0 [72.1; 94.7]	60.0 [32.3; 83.7]	88.1 [74.4; 96.0]
QF-Mg < 1.29 and TTV ≥ 4.27	M1	M1–M4	37.5 [8.5; 75.5]	87.8 [75.2; 95.4]	33.3 [7.5; 70.1]	89.6 [77.3; 96.5]
QF-Mg < 1.29 and TTV ≥ 4.27	M1	M1–M6	45.4 [16.8; 76.6]	91.3 [79.2; 98.6]	55.6 [21.2; 86.3]	87.5 [74.7; 95.3]
QF-Mg ≤ 1.27 and TTV > 4.23	M1	M1–M12	42.9 [17.7; 71.1]	93.0 [80.9; 98.5]	66.7 [29.9; 92.5]	83.3 [70.0; 92.5]

The diagnostic accuracy of the combination of markers was assessed using the optimal QF-CMV and TTV cut-offs determined by ROC curves. Abbreviations: AUC, Area under the ROC Curve; PPV, positive predictive value; NPV, negative predictive value; and CI, confidence interval.

* Calculated in patients with data available over the study period.

For TTV load combined with the QF-Mg at M1, it showed sensitivity of about 40%, and specificity of about 90% (Table 8). NPV were of at least 83%, whereas PPV ranged from 33 to 67%.

## 4. Discussion

In the present study, we evaluated the value of TTV-DNA load and the QF-Ag or the QF-Mg, alone and in combination, for prediction of CMV reactivation (≥ 3 log_10_ IU/ml) during the first post-transplant year. The multicentric observational QuanticR+ prospective cohort of R+ KTR was specifically designed for this purpose. This allowed to recruit a population with homogeneous follow-up and centralized TTV load and QF-CMV evaluation. We then determined new TTV, QF-Ag, and QF-Mg cut-offs that may improve risk stratification of CMV reactivation.

We first investigated TTV load dynamics in the first post-transplant year in our R+ KTR cohort. At D0, most patients had detectable TTV DNAemia and 44.1% had TTV load ≥3.45 log_10_ copies/ml in whole blood. These results are in line with previous reports demonstrating a higher TTV viremia prevalence in KTR than in immunocompetent healthy individuals and TTV reactivation in almost all immunocompromised patients (40–42). TTV load dynamics increased from baseline and reached a peak at M3, which coincides with the period of maximum immunosuppression. Thereafter, TTV slowly declined from M3 to M12. Similar TTV kinetics were already described in various transplant settings (27, 33, 43). In our CMV-seropositive adult population, we also confirm that anti-CMV prophylaxis had no effect on TTV viremia (44).

During the 1-year follow-up, 19 KTR developed CMV reactivation (incidence of 29.7%). This incidence is comparable with previous published results (45). TTV load was higher in KTR with CMV reactivation compared to patients without reactivation throughout the follow-up period. Therefore, we focused on the earliest follow-up time points (D0 and M1) to evaluate the diagnostic performance of TTV load for risk prediction of CMV reactivation. Moreover, we assessed whether the increase in TTV levels between D0 and M1 was significantly associated with a higher incidence of CMV reactivation. According to our results, a TTV load ≥3.45 log_10_ copies/ml at D0 or M1 appears to be an interesting indicator of immunosuppression in patients experiencing CMV reactivation, with sensitivity values ranging from 57 to 79%. Although PPV were less than 50%, TTV load demonstrated high NPV. Therefore, TTV load at D0 or M1 seems better predict a CMV viremia control than a reactivation in R+ KTR during the first year of transplantation. Nevertheless, a significant difference in one-year CMV-free infection rates between patients with a TTV load above or below the 3.45 log_10_ copies/ml cut-off was only obtained at D0.

According to ROC analyses, the optimal TTV cut-off value at D0 was defined as ≥3.97 log_10_ copies/ml to predict CMV reactivation within the following 6 months (D0-M6), and as >3.78 log_10_ copies/ml to predict CMV reactivation within the following 12 months (D0-M12). The optimal TTV cut-offs at M1 were defined as ≥4.27 log_10_ copies/ml for prediction of CMV reactivation within the following 3 or 5 months, and as >4.23 log_10_ copies/ml for prediction of CMV reactivation within the following 11 months. However, AUC values were low, except at D0 for prediction of CMV reactivation within the following 12 months (AUC close to 0.70), and at M1 for prediction of CMV reactivation within the following 11 months (AUC=0.70). Thus, only these two optimized cut-offs may be clinically useful given that the other cut-offs have low diagnostic accuracy. Unlike the 3.45 log_10_ copies/ml cut-off, our optimized TTV cut-off at M1 allowed to reach a statistically significant difference in CMV infection-free survival at M12 between patients with a TTV load above or below the cut-off. In addition, our optimized TTV cut-off at D0 allowed to obtain a higher statistical difference in CMV infection-free survival at M12. Thus, we suggest that R+ KTR with a TTV load >3.78 log_10_ copies/ml at D0 or > 4.23 log_10_ copies/ml at M1 have a significantly increased risk of CMV reactivation during the first post-transplant year.

However, it is important to highlight, that the 3.45 log_10_ copies/ml cut-off proposed by Maggi et al. (27) was defined for plasma samples within the first 10 days post-transplant using an in-house PCR, whereas the quantification of TTV load in the current study was performed in peripheral blood with the TTV R-GENE® kit (bioMérieux, France). A recent review indicates that the 3.45 log_10_ copies/ml cut-off changes to a value of 3.8 log_10_ copies/ml if converted to values corresponding to the commercial PCR we used (21). Interestingly, we were able to find the same TTV cut-off value at D0 (3.78 log_10_ copies/ml), in whole blood. Fernández-Ruiz et al. further reported that a plasma TTV load >4.56 log_10_ copies/ml at M1 (commercial PCR) was associated with a higher risk of immunosuppression-related adverse events, encompassing the occurrence of opportunistic infection, in the first year after kidney transplantation (29). Of note, using the same TTV quantification kit, we determined in whole blood a TTV cut-off at M1 close to that reported by Fernández-Ruiz et al. As a whole, these results indicate that the predictive value of TTV load is robust and can be used in both plasma and peripheral blood samples. Some other studies have defined cut-offs correlating with the development of infections. Using an in-house PCR, Doberer et al. suggested a TTV load in peripheral blood >8 log_10_ copies/ml, quantified after stabilization at the end of post-transplant month 3, as a risk factor of infection in the first year post-transplantation in kidney recipients (30). This cut-off corresponds to 6.6 log_10_ copies/ml if converted to values corresponding to the commercial PCR (21).

We also assessed dynamic changes in TTV levels between D0 and M1 as a predictor of CMV reactivation between M1 to M12. An increase of at least 0.75 log_10_ copies/ml from D0 to M1 was identified as the optimal value. However, the AUC demonstrated a poor discriminatory power between patients with and without CMV reactivation. In addition, it provided comparable diagnostic performance of those estimated with a static measurement of TTV load at D0 and M1 (cut-off of 3.45 log_10_ copies/ml), and consequently conferred no additional benefit.

Reconstitution of CMV-specific T-cell (QF-Ag) and overall T-cell (QF-Mg) responses was also analyzed according to antiviral prophylaxis administration in the 1-year study period. Our data demonstrated that patients receiving prophylaxis had significantly lower mean QF-Ag values between M1 and M6 compared to patients without prophylaxis. In addition, QF-Ag kinetics exhibited a first increase in mean values from M1 in patients without prophylaxis, whereas values only increased from M5 in the prophylaxis group. Conversely, anti-CMV prophylaxis did not show a significant effect on mean QF-Mg values between patients with and without prophylaxis. Valganciclovir and ganciclovir were the most frequently drugs used in prophylaxis regimens. Our results are in line with previous findings reporting a potential impact of ganciclovir prophylaxis and other nucleoside antiviral agents on delayed recovery of HLA-restricted CMV-specific T-cell responses (46–48). Delayed recovery of virus-specific host response in ganciclovir recipients may be associated with suppression of *in vivo* priming and expansion of CMV-specific T-cell precursors, caused by an efficient inhibition of CMV replication during the period of drug administration (46). Impaired immunoglobulin-G seroconversion and inhibition of CMV-specific IgG antibody maturation have been previously described in transplant recipients receiving ganciclovir prophylaxis (49, 50). Interestingly, QF-Ag kinetics in patients with CMV reactivation seemed to show a strongest recovery of CMV specific T-cell responses from M8 to M12 compared to patients without CMV reactivation, suggesting a stimulation of specific immune responses upon CMV antigen re-exposure in R+ KTR.

The QF-CMV kinetics in patients who experienced CMV reactivation showed that QF-Ag and QF-Mg values tended to be lower at M1 but not at D0. Based on these observations, we hypothesized that a pre-transplant QF-CMV was not predictive of CMV reactivation in CMV-seropositive KTR during the first year of transplantation. This is consistent with a previous study conducted by Lee and colleagues, who showed no association between a pre-transplant QF-CMV assay and CMV DNAemia in R+ KTR (51). These findings were confirmed by Pongsakornkullachart and colleagues, that could not demonstrate the value of a pre-transplant QF-CMV as a predictor for post-transplant CMV viremia in R+ KTR (52). Thus, we focused on a QF-Ag or a QF-Mg at M1 as a relevant time-point for risk prediction of CMV reactivation within the following 3, 5, and 11 months.

The primary analysis was performed using manufacturer’s cut-offs. Although the QF-Ag or the QF-Mg at M1 were poorly sensitive for prediction of CMV reactivation, they appear to be correlated with protection against CMV reactivation. Predictive values indicated that a reactive QF-Ag or QF-Mg at M1 exhibited better performance to predict CMV viremia control than a non-reactive result to predict CMV reactivation in R+ KTR. However, no significant difference in 1-year CMV infection-free rates was found between patients with a reactive and a non-reactive QF-Ag at M1. Thus, a QF-Ag < 0.2 IU/ml at M1 does not predict a significantly higher risk of CMV reactivation at M12 than a QF-Ag above this cut-off in our R+ KTR cohort. CMV-specific T-cell responses (QF-Ag) does not appear to be a powerful marker for assessing the risk of CMV reactivation in our cohort. In contrast, patients with a QF-Mg < 0.5 IU/ml at M1 had a subsequent higher incidence of CMV reactivation at M12 than patients with a QF-Mg > 0.5 IU/ml. Taken together, these results support a better predictive value of a QF-Mg < 0.5 IU/ml than a QF-Ag < 0.2 IU/ml at M1 in R+ KTR. Thus, global T-cell responses (QF-Mg) appear to be more effective in stratifying the risk of CMV reactivation than CMV-specific T-cell responses (QF-Ag) in our R+ KTR cohort. Non-response to mitogen has already been reported as a potential marker of global immunosuppression, suggesting that global T-cell anergy is at high risk for CMV infection or disease (8, 12, 53). A study in 124 D+/R+ SOT recipients showed that patients with an indeterminate QF-CMV result (QF-Ag < 0.1 IU/ml and QF-Mg < 0.5 IU/ml) had a significantly higher risk of CMV disease than those with a non-reactive result (QF-Ag < 0.1 IU/ml and QF-Mg ≥ 0.5 IU/ml) (8). In a study of CMV-seropositive heart transplant recipients, a higher proportion of patients with an indeterminate QF-CMV result (QF-Ag < 0.2 IU/ml and QF-Mg < 0.5 IU/ml) after the suspension of prophylaxis developed a post-transplant CMV infection compared to patients who showed a global T-cell responsiveness (53). Another study performed on 25 KTR showed that patients with an indeterminate QF-CMV or a QF-Mg < 3.5 IU/ml (cut-off used in this study) had an increased incidence of CMV disease or serious infectious complications (15).

Based on ROC curve analyses, we determined optimized QF-Ag and QF-Mg cut-offs at M1 for prediction of CMV reactivation. QF-Ag cut-off values at M1 were defined as <2.23, <9.12, and ≤2.48 IU/ml for CMV reactivation within the following 3, 5, and 11 months, respectively. As suggested by Pongsakornkullachart et al. (52), the QF-Ag cut-off of 0.2 IU/ml (as per manufacturer’s instructions) may be appropriate only for CMV-seronegative recipients and higher cut-offs might be required in KTR previously exposed against CMV. Other studies have already reported high QF-Ag cut-offs associated with the absence of CMV infection in SOT. Gliga et al. defined a QF-Ag cut-off of 85.1 IU/ml for prediction of protection from CMV viremia within 3 months following the QF-CMV assay (54). Another study assessing the risk of CMV reactivation in post-bone marrow transplant patients suggested a cut-off of >8.9 IU/ml for protection from high-level CMV viremia and CMV disease (55). In a study conducted by Abate et al., QF-Ag cut-offs of >1 to 6 IU/ml were associated with protection from CMV infection in KTR (56).

Subsequently, we calculated the performance of the QF-Ag at M1 with our optimized cut-offs. Better sensitivity values were found. To be noted, the gain in sensitivity with these optimized cut-offs resulted in a loss of specificity. Predictive values were overall similar with those obtained with manufacturer’s cut-offs. Regarding the AUC, they yielded poor results to predict CMV infection within the following 3, 5, or 11 months. Moreover, the QF-Ag cut-off of 2.48 IU/ml did not allow to reach a statistically significant difference in CMV infection-free survival at M12 between patients with a reactive and a non-reactive QF-Ag at M1. Similarly, to a QF-Ag < 0.2, a QF-Ag ≤ 2.48 IU/ml at M1 does not predict a significantly higher risk of CMV reactivation than a QF-Ag above this cut-off.

The diagnostic accuracy of the QF-Mg at M1 was also evaluated using cut-offs determined by ROC curves. In a similar way as for the QF-Ag, it resulted in an increase in sensitivity, at the detriment of specificity. Predictive values were overall similar with those obtained with manufacturer’s cut-offs. The QF-Mg at M1 showed a poor discriminatory power to predict CMV infection within the following 3 months. Conversely, ROC curve analyses revealed 1.29 IU/ml, and 1.27 IU/ml as accurate QF-Mg cut-offs allowing to discriminate patients with and without subsequent CMV reactivation within the following 5 and 11 months, respectively. Moreover, Kaplan–Meier analyses demonstrated that the 1.27 IU/ml cut-off better stratify the risk of CMV reactivation at M12 than the manufacturer’s QF-Mg cut-off in our R+ KTR cohort. Further studies are warranted to reevaluate the potential use of this QF-Mg cut-off for risk stratification of CMV reactivation during the first year of transplantation in R+ KTR. As a whole, this study demonstrates the limited potential of the QF-Ag compared to the QF-Mg in stratifying risk of reactivation in R+ KTR. Previous studies in transplant recipients have shown that other assays, such as ELISpot or flow cytometry, perform better for this purpose (57, 58). Furthermore, as the QF-CMV uses certain HLA restricted CMV peptides, patients with HLA types not covered by this assay might not be detectable notwithstanding the actual presence of CMV-specific T cells. Lack of these HLA alleles could be an explanation for some false-negative (patients with a negative QF-Ag but no CMV reactivation) in our study.

Our results show that manufacturer’s cut-offs should be refined to better adapt prediction in CMV-seropositive populations. We then hypothesized that combining markers for specific and global immune response evaluation could enhance their predictive values. We thus analyzed the value of TTV load in combination with the QF-Ag or the QF-Mg at M1 for risk prediction of CMV reactivation within the following 3, 5, and 11 months. Using conventional cut-offs, the combination of TTV load and the QF-Ag or the QF-Mg at M1 did not outperform the negative predictive values as compared with TTV load, the QF-Ag or the QF-Mg analyzed independently. Nevertheless, the combination of TTV load and QF-Ag or TTV load and QF-Mg resulted in an increase of positive predictive values. By using cut-offs obtained from ROC curves, we overall further increased positive predictive values, and therefore, slightly improved prediction of patients at risk for CMV reactivation.

Our study has some limitations. Although anti-CMV prophylaxis has been shown to significantly decrease QF-Ag values between M1 and M6, we could not conduct the analysis separately in the subgroups of patients with and without prophylaxis. Indeed, the number of patients in each subgroup was too small to assess the risk of post-transplant CMV reactivation. In addition, the number of episodes of CMV reactivation was low, limiting the statistical power of the subgroup analysis.

In conclusion, TTV load or the QF-Ag or the QF-Mg did not predict subsequent CMV reactivation (≥ 3 log_10_ IU/ml) in our R+ KTR cohort but rather identified patients at low risk of CMV reactivation. Global immunity (TTV load or QF-Mg) appears to perform better than specific anti-CMV immunity (QF-Ag) in stratifying risk of CMV reactivation in our cohort. We propose new TTV cut-offs at D0 and M1 and QF-Mg cut-offs at M1 that could be useful for optimal management of immunosuppressive strategy and for assigning R+ KTR to groups at lower risk of CMV reactivation during the first post-transplant year. Interestingly, the combination of TTV load and QF-Ag or TTV load and QF-Mg seems to improve positive predictive values, and thus improve risk prediction of CMV reactivation. The use of these new cut-offs and this combination may be useful in clinical practice, and merits further validation in larger prospective studies.

## Data availability statement

The raw data supporting the conclusions of this article will be made available by the authors, without undue reservation.

## Ethics statement

The studies involving human participants were reviewed and approved by Ethics Committee of Centre Hospitalier Universitaire de Limoges. The patients/participants provided their written informed consent to participate in this study.

## Author contributions

SA and ME conceived and design the study. SA was the primary investigator of the QuanticR^+^ clinical study. ME was clinical coordinator of the QuanticR^+^ study. SA and FG organized the database. MG-M performed the centralized analysis for TTV and CMV viral load. SM, FG, SH, and SA validated the data base. AL performed the statistical analysis. SM, SA, and SH reviewed data analysis. SM wrote the first draft of the manuscript and implemented all the revisions. SA, SH, and AL wrote sections of the manuscript and reviewed the manuscript. Other authors included patients in the cohort and provided data and are cited by the number of included patients. All authors contributed to the article and approved the submitted version.

## Funding

This work was supported by local grant from the Clinical Research department of the Centre Hospitalier Universitaire de Limoges, bioMérieux for providing the TTV assay kits and Qiagen for discount in Quantiferon assays reagents costs.

## Conflict of interest

The authors declare that the research was conducted in the absence of any commercial or financial relationships that could be construed as a potential conflict of interest.

## Publisher’s note

All claims expressed in this article are solely those of the authors and do not necessarily represent those of their affiliated organizations, or those of the publisher, the editors and the reviewers. Any product that may be evaluated in this article, or claim that may be made by its manufacturer, is not guaranteed or endorsed by the publisher.
