# Supplementary material for: Torque teno virus viremia and QuantiFERON®-CMV assay in prediction of cytomegalovirus reactivation in R+ kidney transplant recipients
Source: Front Med (Lausanne). 2023 Jun 22;10:1180769. doi: 10.3389/fmed.2023.1180769 (PMC10323437; doi:10.3389/fmed.2023.1180769)
Supplement: Supplementary file 1 [file Data_Sheet_1.docx]

Supplementary Material

# Supplementary Figures

**Figure S1. Flow chart of the QuanticR+ study participants.**

#

**(A)**

**(B)**

**(C)**

**(D)**

**(F)**

**(E)**

**Figure S2. Receiver operating characteristic (ROC) curve and their relative AUC to distinguish the optimal TTV cut-off value at D0 for prediction of CMV reactivation between D0 and M6 (A); D0 and M12 (B), the optimal dynamic change in TTV levels between D0 and M1 for prediction of CMV reactivation between M1 and M12 (C), and the optimal TTV cut-off value at M1 for prediction of CMV reactivation between M1 and M4 (D); M1 and M6 (E); M1 and M12 (F).**

*Abbreviations: AUC, area under the curve.*

**(F)**

**(E)**

**(D)**

**(C)**

**(B)**

**(A)**

**Figure S3. Receiver operating characteristic (ROC) curve and their relative AUC to distinguish optimal cut-off values of the QF-Ag (A, B, C) and the QF-Mg (D, E, F) at M1 for prediction of CMV reactivation between M1 and M4 (A, D); M1 and M6 (B, E); M1 and M12 (C, F).**

*Abbreviations: AUC, area under the curve.*

**
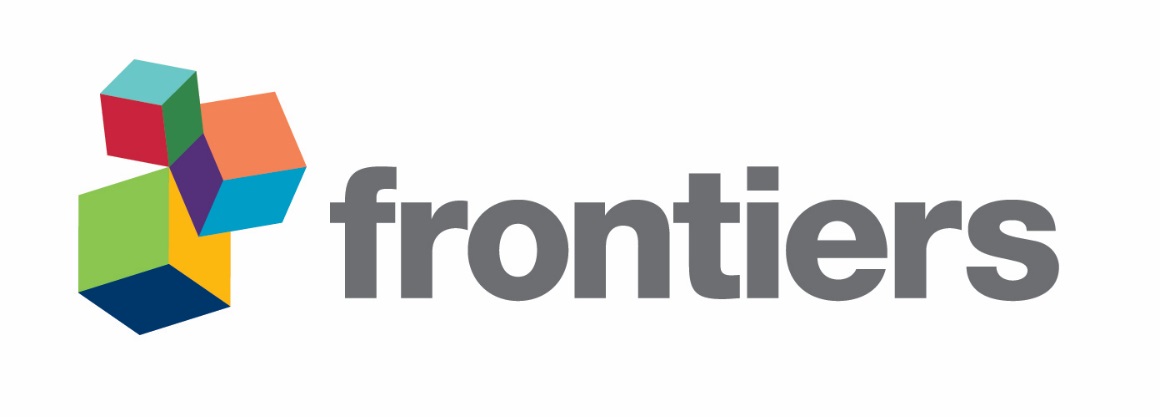
**
